# Supplementary material for: Cell-type-specific resolution epigenetics without the need for cell sorting or single-cell biology
Source: Nat Commun. 2019 Jul 31;10:3417. doi: 10.1038/s41467-019-11052-9 (PMC6668473; doi:10.1038/s41467-019-11052-9)
Supplement: Supplementary file 4 — Description of Additional Supplementary Files [file 41467_2019_11052_MOESM4_ESM.pdf]

Description of Additional Supplementary Files

File Name: Supplementary Data 1
Description: Results of differential methylation in immune activity

File Name: Supplementary Data 2
Description: Results of differential methylation in rheumatoid arthritis
